# Prototype of Hydrochemical Regime Monitoring System for Fish Farms

**DOI:** 10.3390/s26020497

**Published:** 2026-01-12

**Authors:** Sergiy Ivanov, Oleksandr Korchenko, Grzegorz Litawa, Pavlo Oliinyk, Olena Oliinyk

**Affiliations:** 1 Educational-Scientific Institute of Telecommunications, State University of Information and Communication Technologies, 03110 Kyiv, Ukraine; 2 The Institute of Security and Information Technology, University of the National Education Commission, 30-084 Krakow, Poland; oleksandr.korchenko@uken.krakow.pl (O.K.); grzegorz.litawa@uken.krakow.pl (G.L.); 3 Faculty of Physics and Mathematics, National Technical University of Ukraine “Igor Sikorsky Kyiv Polytechnic Institute”, 03056 Kyiv, Ukraine; poleinik@ukr.net; 4 Institute of Fisheries of the National Academy of Agrarian Sciences of Ukraine, 03164 Kyiv, Ukraine; elenaoli@ukr.net

**Keywords:** aquaculture, long-range wide-area network, hydrochemical regime modeling, extended Kalman filter, predictive monitoring, fish kill prediction

## Abstract

This paper presents a prototype of an autonomous hydrochemical monitoring system developed for large freshwater aquaculture facilities, directly addressing the need for smart monitoring in Agriculture 4.0. The proposed solution employs low-power sensor nodes based on commercially available components and long-range LoRaWAN communication to achieve continuous, scalable, and energy-efficient water quality monitoring. Each sensor module performs on-board signal preprocessing, including anomaly detection and short-term forecasting of key hydrochemical parameters. An ecological pond dynamics model incorporating an Extended Kalman Filter is used to fuse heterogeneous sensor data with predictive estimates, thus increasing measurement reliability. High-level data analysis, long-term storage, and cross-site comparison are performed on the server side. This integration enables adaptive tracking of environmental variations, supports early detection of hazardous trends associated with fish mortality risks, and allows one to explain and justify the reasoning behind every recommended corrective action. The performance of the forecasting and filtering algorithms is evaluated, and key system characteristics—including measurement accuracy, power consumption, and scalability—are discussed. Preliminary tests of the system prototype have shown that it can predict the dissolved oxygen level with RMSE = 0.104 mg/L even with a minimum set of sensors. The results demonstrate that the proposed conceptual design of the system can be used as a base for real-time monitoring and predictive assessment of hydrochemical conditions in aquaculture environments.

## 1. Introduction

Global climate change causes unstable and abnormal weather, which mostly manifests itself as long periods of excessive heat and as a rapid change in temperature. Because of sudden temperature fluctuations, the hydrochemical regimes of fish farm ponds may become worse, and that may lead to fish kills. In order to prevent economic losses related to the mentioned phenomenon, prediction of the hydrochemical regime in the pond can be used. The hydrochemical regime of the pond is determined by the parameters of its water [1], in the first place by the temperature, dissolved oxygen (DO), and pH. In most fish farms, hydrochemical parameters are measured with a predefined period [2]. At that, all measurements are performed either using mobile testing systems for express measurement [3] or simply collecting test samples of the water and then analyzing those samples in the specialized laboratory [1]. Such an approach has some drawbacks:

- Data collection in both cases usually takes a significant time because it is performed by “a man in the field”.
- Analysis of collected samples using non-express chemical methods also requires some time to implement and obtain correct results.

As a result, data processing and analysis is performed with a time lag, and any dangerous phenomena may manifest themselves well before any data are analyzed and any decision is made. The use of an online hydrochemical regime condition monitoring system allows one to eliminate those drawbacks, thus allowing for early detection of possible problems, including fish kills. Use of such an online monitoring system, built using the concept of smart farming [4], may significantly decrease farm losses and increase fish farm productivity.

The Internet of Things (IoT) is a very promising technology for use in smart agriculture and especially in fish farming. IoT allows one to build a non-centralized network for data measurement and collection, in which each measurement module (node) is independent of the others. IoT nodes may utilize different communication technologies and protocols, allowing for automated identification of modules and control of remote objects (e.g., aerators or water discharges) [5]. Thus, data collection from different sensors, data transmission through wireless communication networks, and data processing can now be implemented on different levels of a monitoring system [6]. A key feature of an IoT-based measurement module is its ability to implement basic data processing and measurement in one place, as stated, e.g., in [7]; that allows one to reduce data flow in the system and thus improve its reliability. Drawbacks of IoT include limited computing power of the module (which limits data processing to a set of simple operations) and possible cybersecurity issues, especially when a public network is used for data transmission and data are stored on the servers available to the public.

In recent years, many fish pond monitoring systems have been developed, based on distributed monitoring and data collection, unmanned air vehicles (UAVs), and on Internet of Things solutions. In [8], a general review of IoT technologies in smart farming is given; those technologies include Wi-Fi (Wireless Fidelity), Bluetooth, ZigBee, and Long Range (LoRa). Manoj et al. [9] made a similar review of various water quality monitoring systems that have been proposed by various researchers in 2011–2020. Analysis of the recent works, related to the use of IoT in fish farming, is presented below.

### 1.1. Related Works

Chatziantoniou et al. [10] proposed a monitoring tool, called “Aquasafe”, which was evaluated for its effectiveness and performance by test users through real-life scenarios. That system is developed for use in marine aquaculture; the main water parameters used were dissolved oxygen (DO) and sea surface temperature. Some of those parameters were to be measured from UAVs. Empiric mathematical models were used for prediction of values of those parameters, and based on the predicted values, the state of aquaculture was predicted.

In [11], Akhter et al. proposed a system based on static modules. Those modules are powered by solar cells and provide temperature, pH, nitrate, phosphate, calcium, magnesium, and DO measurement. A number of such modules may be used as a base of IoT cloud. A strong side of the study is the discussion of critical water parameters (temperature, pH, nitrate, phosphate, calcium, magnesium, and dissolved oxygen), along with a review of the sensors available. However, the authors of [11] do not provide any estimation of the system precision.

Khudoyberdiev et al. [12] proposed an IoT-based predictive optimization approach for efficient control and energy utilization in smart fish farming. Fuzzy logic is used to calculate control parameters for IoT actuators, which allow one to provide predicted optimal water quality parameters while minimizing energy consumption. Parameters measured and controlled are temperature, pH, water, and electric conductivity levels.

Al-Mutairi and Al-Aubidy [13] proposed an IoT-based system for real-time monitoring, control, and management of fish farming. Each pond is controlled by one embedded microcontroller; pH, turbidity, temperature, DO, electric conductivity, total dissolved solids (TDS), and water level are measured, along with air temperature and light. Corrective measures are taken based on the decision made by the fuzzy controller; those measures include water inlet control, drainage control, oxygen supply control, air-cooling, fans, exhaust, and light control. As a drawback of such a system, one should note that the use of all those measures but oxygen, water inlet, and drainage control are impractical in real-life scenarios, where pond area may be as high as tens of hectares. Moreover, no pH correction is performed, and pH is a critical water parameter.

Mohd Jais et al. [14] in their work developed a monitoring system for Asian seabass fish farming in aquaculture tanks. Water temperature, DO, pH, ammonia, and salinity were measured using low-cost sensors, aimed on Arduino usage. The accuracy of those sensors was enhanced using simple linear regression; as a system base, a Virtuino application and ESP8266 Wi-Fi module were used. The main drawbacks of such an approach are the small signal radius of Wi-Fi signal and the use of laboratory-grade sensors that renders such a system ineffective for monitoring of large ponds.

Mat Tahir et al. [15] proposed a similar system targeting tank-based aquaculture. That system measures pH and provides fish feeding using an automatic feeder with food level measurement. Tolentino et al. [16] proposed a monitoring and correction system for intensive aquaculture based on Arduino and Raspberry Pie, using Long-Range Wide-Area Network (LoRaWAN) as a data transfer technology. Temperature, pH level, oxidation–reduction potential, turbidity, salinity, and DO were to be measured and controlled in tanks using that system. All those systems were also designed for use in fish tanks, and as such cannot be effectively used on large-area ponds.

Chen et al. [17] proposed an automated system for monitoring of pond water quality. The set of the sensors is basic and includes a temperature sensor, pH meter, DO sensor, and water level sensor. However, use of the pH meter, that cannot be submerged for a long time, led to the use of a programmable logic controller (PLC)-controlled robotic arm that provides automatic measurement and sensor maintenance. That significantly decreases energy efficiency of the system and makes its use and maintenance more complex.

Tamim et al. [18] presented another IoT water quality monitoring system that monitors pH and temperature; additional parameters such as DO and ammonia are estimated using testing kits. An Android-based application is used to send measured data to the users; the drawback of the systems is use of Wi-Fi for data transfer. That means limited transmission range and high sensitivity to obstacles and weather.

Similar systems are now developed for wastewater treatment. For example, Salem et al. [19] proposed and tested an IoT system for wastewater management, which collects data about pH and temperature of wastewater and informs users about unexpected industrial wastewater inlets via SMS notification; the system may also control the valves of the plant. The system proposed is based on ESP8266 board and stores data in a cloud but does not provide any modeling or decision support. A similar system, that uses turbidity, TDS, temperature, and humidity sensors, is presented by Valarmathi et al. in [20]; collected data are transmitted for further processing via Wi-Fi to a dedicated server rather than cloud.

Chavan et al. [21] in their wastewater monitoring system APAH used pH, DO, electrical conductivity, TDS, turbidity, and temperature sensors to estimate water state. Machine learning was used in order to provide decision support for wastewater plant personnel. The APAH system is based on ESP8266 board and uses Wi-Fi and cellular networks for data transmission.

General features of all the systems mentioned above are shown in Table 1.

As one can see from the analysis made above and Table 1, hydrochemical regime monitoring systems for fish farming are now in the active research and development phase. However, a majority of the systems already developed are aimed at small farms or tank aquaculture, and that does not allow one to use such systems on large fish farm ponds. That is caused by the following:

- systems that target fish tanks or small farms cannot cover large-area ponds and adequately estimate the hydrochemical regime of the pond with an area of tens of hectares;
- systems that use Wi-Fi (like the one proposed in [18]) have limited range between modules due to the nature of the Wi-Fi signal, which is sensitive to obstacles;
- cellular-based systems require monthly payment for use of telecommunication company services and protection of data and protocols in order to ensure cybersecurity.

The “Aquasafe” system targets marine aquaculture and thus may cover large water areas of an industrial fish farm. The mentioned system takes algal blooms, sea surface temperature, fish growth, and wind speed/gusts into account using satellite imagery. However, it does not measure other important water parameters (like DO and pH) online and thus cannot be used in freshwater aquaculture without modification. One should also note that the use of UAVs for data collection requires significant costs, and the use of the Web for data transfer requires data protection.

### 1.2. Goal of This Study

The authors of this paper propose combining an IoT-based approach to data collection and transmission with well-established principles of condition monitoring in one system. That will allow the use of readily available components, thereby simplifying system development and reducing its cost while ensuring low energy consumption and high autonomy of the measurement modules.

In addition, the fish pond ecosystem model is employed to predict pond parameters, and the Extended Kalman Filter (EKF) is used to allow the model to adapt to changes in pond conditions based on the measured data. Application of the EKF improves the overall reliability of the system and allows long-term forecasting (up to 20–30 days). Such an approach also allows one to explain and justify the reasoning behind every recommended corrective action for the ponds. Therefore, any decision regarding fish farm operation made by, or with the assistance of, the monitoring system can be fully interpreted and justified—something that is not possible, for example, when neural networks are used.

To the best of the authors’ knowledge, this approach has not been applied before. An important feature of the proposed system is its focus on large fish farms, primarily freshwater ones.

**Figure 1:**
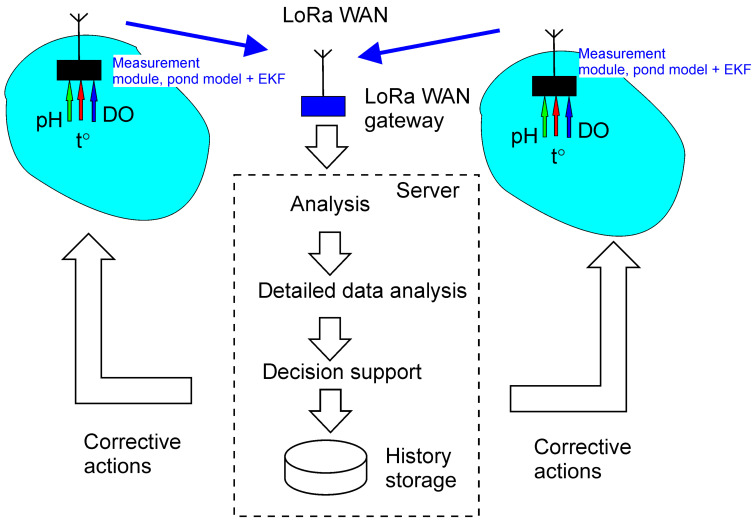
General concept of the monitoring system. Data are collected and pre-processed by measurement modules then sent via LoRaWAN gateway to a server for analysis and initiation of corrective actions.

In Figure 1, the general concept of the system is shown. The monitoring system is based on a network of autonomous measurement modules, which are installed at the ponds of a fish farm. Measurement modules provide operative control of the water conditions in the pond, measuring DO, pH, and water temperature, and employs a pond model with EKF for adaptation to pond conditions. Measured and pre-processed data are transmitted by air to a LoRaWAN gateway and then to a local server, on which dedicated software is running; that software includes the LoRaWAN network server, database, and client software. Based on estimated hydrochemical parameters and prognosis, corrective actions are recommended. Optionally, the system may contain actuator controllers that allow it to initiate and apply corrective actions to the monitored ponds.

The goal of this study is to develop a monitoring system for large-area fish ponds; at that, the system should meet the following requirements:

- long distance between nodes (measurement units)—up to 5–10 km;
- low energy consumption;
- 24/7/365 operation;
- measured parameters should include at least temperature, pH, and DO.
- long period between sensor maintenance and/or replacement (at least 6 months);
- good scalability (ones to tens of nodes);
- automated prognosis of dangerous phenomena in a pond, and recommendation (or initiation) of corrective measures.

The system should integrate physical and chemical measurements with the ecological model in order to estimate non-observable state variables. Complex quantitative estimation of accuracy, stability, and practical feasibility of the system should be presented.

## 2. Materials and Methods

As a theoretical base of this study, well-known general principles of predictive condition monitoring are used. Parameters that should be measured in order to estimate the hydrochemical regime in a fish pond are selected based on [1,2,3]. Outlier elimination and least square approximation are used in order to provide measured data approximation and prediction of parameters levels. Based on those levels, water quality deterioration and, respectively, probable fish kills are predicted.

In order to improve overall reliability of the system, the pond ecosystem is modeled using the Lotka–Volterra and Michaelis–Menten equations. The model explicitly includes three trophic levels: phytoplankton, zooplankton, and fish. Extended Kalman Filter (EKF) is employed to estimate the system state and to predict its behavior based on a limited set of measured data: DO and chlorophyll-a concentration. DO is measured using an optical sensor once an hour, while chlorophyll-a is sampled at a lower frequency (once every three days). System observability is formally analyzed using Lie derivatives. Validation of the proposed algorithm is performed in two distinct stages: initially, the algorithm is validated using a restricted dataset that mimics limited information availability; subsequently, validation using additional, comprehensive field data is planned. The initial validation presented in this work is based on a continuous 30-day monitoring period.

In order to implement condition monitoring, a distributed measurement system is to be used. As a base for the system under development, a network of autonomous modules, which provide parameter measurement and initial data processing, is used. Measured and processed data are transmitted to the server and stored in the database. Dedicated software, which runs on the same server, provides approximation and prediction and, optionally, initiates corrective actions. Information about the current state of the farm may be made available via the Internet.

The network of measurement modules is based on LoRaWAN technology. That allows one to obtain long distances between modules—up to 5–15 km between module and gateway in rural, clear-sky conditions within a line of sight. Use of LoRaWAN also allows one to provide low energy consumption, as modules may be active only during measurements and measured data transmission.

Preliminary tests (functional validation) of the system concept and algorithms were performed during initial field trials in the Kyiv Reservoir (50° $38^{\prime }$ $25.5^{\prime \prime }$ N, 30° $26^{\prime }$ $42.2^{\prime \prime }$ E). The probe was positioned at a 20 cm depth. This horizon is characterized by the highest amplitude of diurnal variation in dissolved oxygen and temperature. This specific deployment depth was chosen to test system functionality under a highly dynamic signal regime and to address practical requirements: ensuring sensor safety, providing a stable environment for calibration consistency, and facilitating logistical execution during preliminary experiments. For measurements, an Atlas Scientific Gen 3 Industrial D.O. Probe sensor [22] with an EZO^TM^ board was utilized. The sensor was calibrated according to the manufacturer’s specifications. System control, preliminary data processing, and logging were managed by a WeAct Black Pill board featuring an STM32F401CEU6 microcontroller. The hardware assembly was secured inside an IP68-rated waterproof case, with only the sensing probes and their cables exposed. Chlorophyll-a was measured using an ATO Chlorophyll Sensor for Water Quality [23] and used for EKF adjustment and during offline algorithm validation. Obtained DO data were processed offline using MATLAB R2016b.

## 3. Basics of Hydrochemical Regime Monitoring in a Fish Pond

Fish kill, depending on its intensity, may lead to death of from 20–30% up to 70–80% of the fish in the pond [1]. The occurrence of a fish kill can be predicted using (but not limited to) the following parameters: water temperature, DO, and pH [1]. Thus, if one measures at least the parameters mentioned, one can predict their change with time and, therefore, the possibility of a fish kill. Based on that prediction, corrective measures (e.g., aeration) may be taken.

Predictive condition monitoring is based on two principles:

- state of an object is related to one (or a combination of some) physical parameter that can be measured and expressed in quantity;
- object state changes with a limited speed, and thus it may be predicted using a set of previously measured data and approximation.

In order to select parameters measured by the hydrochemical regime monitoring system, both references [1,2,3] were used. A general set of water parameters is presented in [3], but no recommendations on the measurement period and use of those parameters for water condition prediction are made in that book.

According to recommendations of “Collection of normative and technical documentation on commercial fish farming” [2], operative control of pond water parameters must include water temperature, DO, and pH in summer and water temperature and DO in winter. Such measurements should be made daily.

The key water parameter for fish health is dissolved oxygen. A detailed survey of its role and the range of tolerance to DO in fish is presented in [2,24]. For carp, grass carp, silver carp, and other carp fish [2], in summer, the desirable DO level should be not less than 5.0 mg/L. If the DO concentration is 3.0 mg/L or less, the condition of the fish deteriorates, and fish cannot feed and grow. When DO concentration is 0.5–0.1 mg/L, the fish dies. In wintering ponds, the dissolved oxygen content should not be less than 6.0 mg/L. For other fish species, DO levels at which respiratory depression and fish death occur according to [25] are shown in Table 2.

One should note that if the DO level is 10 mg/L or higher in the surface layers of water [2], in most cases that means that water is not mixed in a pond, and one has to mix the water in order to break stratification and prevent fish kill.

The next important water parameter is its temperature. In order to determine the presence of stratification in the pond, one should measure the temperature near its surface and near its bottom. Water temperature should be controlled in characteristic points of the pond—near the water inlet, near the discharge, and at the middle of the pond. Moreover, DO and pH levels depend on water temperature, so it has to be measured along with those parameters in order to predict changes of the hydrochemical regime.

pH level is very important for fish health. A normal pH level for carp ponds is 6.5–8.5, but during a day, the pH value may change by 2–3 units. If intensive phytoplankton development occurs (or in thickets of algae and other macrophytes), pH level may take a value of 10–11, which is dangerous to fish, as it causes damage to gills and skin [1,2]. The magnitude of the pH change allows one to draw conclusions about the dynamics of DO concentration in the water. Temperature stratification causes the formation of zones with low oxygen content in the bottom layers of the water. The destructive processes of organic matter and accumulation of substances harmful to fish—ammonia, nitrites, hydrogen sulfide, dominate those layers while the pH decreases. If the ratio of the pH level near the water surface and near the pond bottom in a small pond is higher than 1.05 [1], fish kill is very probable.

Summing up what was stated above, in order to predict the occurrence of fish kill accurately, one has to:

- Constantly monitor water temperature, dissolved oxygen content, and hydrogen index (pH) in the bottom and surface layers of the pond [1]. The monitoring period in summer should be 1 h from 9 p.m. until 6 a.m. and is not strictly defined from 6 a.m. to 9 p.m.; in winter, the monitoring period may be increased.
- Determine timely the boundaries of stratification zones in the reservoir (feeding areas, pits, bottom channels, deep places, etc.).

What is stated above can be assumed to be the base of a hydrochemical regime monitoring system for the fish pond. Other water parameters—including its turbidity, phytoplankton development, CO_2_ level, ammonia, ammonium, nitrite, nitrate, etc.—according to normative and technical documentation recommendations are to be measured once every 10–15 days [2]. Taking the cost of the sensors needed and the required sampling interval into account, it is more cost effective to measure those parameters using the mobile monitoring system rather than an online one. More thorough tests that include other water parameters should be performed by the specialized laboratory, as those tests are used rarely.

## 4. System Structure and Design

### 4.1. Sensor Selection

The base of every monitoring system is its sensors. Taking all said above into account, sensors for hydrochemical regime monitoring in a fish pond should meet the following requirements:

1. sensors must operate underwater at depths up to 10–20 m (pond depth of 50 m and more is very rare);
2. sensors must operate underwater for long periods—preferably for months;
3. sensors should have a time before re-calibration of at least 6 months—the more the better;
4. sensors should have a service period of at least 6 months—the more the better.

All requirements stated above allow one to ensure continuous system operation for a desired period of 6 months at least, which is determined by the technological cycle of the farm in temperate climate (fish wintering). With such a period, maintenance of sensors and measurement modules will be performed while fish are moved from one group of ponds into another one and will not affect either fish condition or normal farm operation. That should greatly increase system effectiveness and decrease its maintenance and exploitation costs.

Most Arduino-oriented sensors, like the ones used in [14], do not meet the above requirements; a detailed comparison of some of the industrial-grade and laboratory-grade sensors available on the market that can be used for hydrochemical regime monitoring in a fish pond is presented in Appendix A.

From the analysis presented in Appendix A it follows that in most cases, i.e., for fish farming of common river fish like carp, the use of Atlas Scientific or similar sensors with their relatively low retail price is preferable.

If a fish farm is dedicated to trout or sturgeon, sensors that are more expensive could be used. In addition to DO and pH sensors, CO_2_ level, ammonia, and other sensors may be used in order to improve monitoring quality. To that end, the use of standard interfaces to such sensors is mandatory, and the software of the system will have to be changed.

### 4.2. System Structure

**Figure 2:**
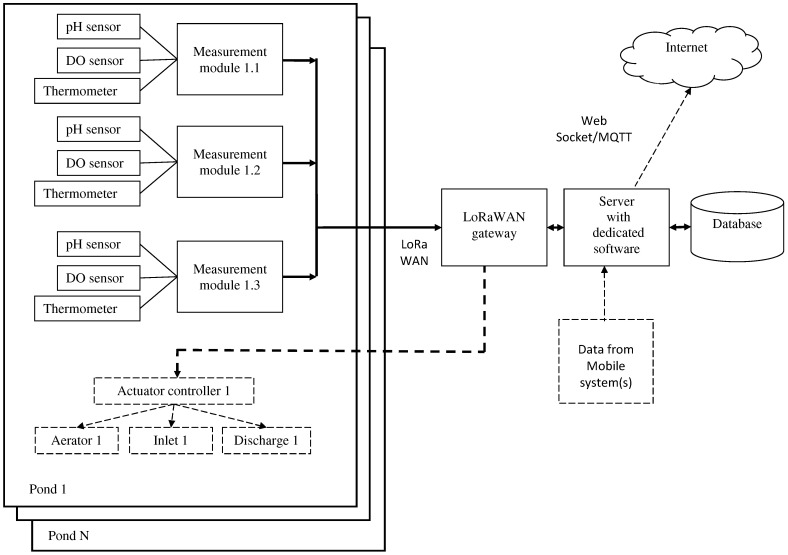
Structure of the proposed system. Optional parts are drawn in dashed lines; additional data from mobile monitoring systems may be used in order to improve prognosis.

The structure of the proposed system is shown in Figure 2. The data flow is shown as lines with arrows; optional components and data flows are drawn with dashed lines.

As one can see, the monitoring system consists of some autonomous measurement modules, which are installed at the ponds of a fish farm, a LoRaWAN gateway, and a server with database and dedicated software. Optionally, the system may contain actuator controllers, which allow it to make corrective actions on the monitored ponds. In most cases, those controllers have to provide simple functions, like start and stop of the aerator, closing and opening inlet or discharge, etc.

Water condition should be measured in at least three points of the pond, mentioned above. In the case of a large pond area, there may be some additional characteristic points for water parameter measurement: feeding areas, pits, bottom channels, and deep places are some of the examples. Therefore, the number of measurement modules per pond in each and every case should be determined individually.

Measured data are transmitted via LoRaWAN gateway to a local server, on which dedicated software is running; that software includes the LoRaWAN network server, database, and client software. A detailed description and functioning of the software is given below. The data obtained may be made accessible via Internet connection using Web Socket or Message Queue Telemetry Transport (MQTT). The LoRaWAN network of the proposed system has star topology; in the case when the area is very large, daisy chain topology may be used instead.

All data analysis and decision making described in Section 4.4 can be performed on the central server. However, in order to increase system reliability, it is preferable to distribute data processing between system nodes. Measurement modules may perform basic data processing, including outlier detection and prognosis of water parameters in the measurement point. On the base of data obtained, modeling of the hydrochemical regime of the pond can also be performed on each node of the system; the model of the regime may be based on differential equations or neural network. More thorough data processing, including comparative analysis of obtained and history data and storage of data history should be performed on the server. Additional data from the mobile testing system may also be used in order to increase reliability of hydrochemical regime prediction. Such an approach allows one to decease data flow in the system; moreover, even if one or some of the modules fail, the monitoring system will continue operate as a whole. At that, the final data processing has to be provided by custom client software.

Based on data processing performed by client software, corrective actions may be initiated. In the simplest case, that is characterized by giving recommendations to service personnel; if optional actuator controllers are used, some of the measures may be applied automatically. The modeling, data processing, and decision-making process is described below.

### 4.3. Modeling of the Hydrochemical Regime of the Pond

A pond ecosystem in its basic form contains phytoplankton, zooplankton, and fish. Therefore, an analytical model of a pond ecosystem, based on Lotka–Volterra and Michaelis–Menton equations [26], is defined by the following system of ordinary differential equations:

(1) $$\left\{\begin{array}{c} \frac{dP}{dt}=r_{P}P\left(1-\frac{P}{K}\right)-g_{Z}\frac{PZ}{h_{P}+P}-m_{P}P,\\ \frac{dZ}{dt}=e_{Z}g_{Z}\frac{PZ}{h_{P}+P}-g_{F}\frac{ZF}{h_{Z}+Z}-m_{Z}Z,\\ \frac{dF}{dt}=e_{F}g_{F}\frac{ZF}{h_{Z}+Z}-m_{F}F,\\ \frac{dO}{dt}=a_{P}P-b_{R}\left(P+Z+F\right)+k_{2}\left(O_{sat}-O\right), \end{array}\right.$$

where *P*, *Z*, *F* are biomasses of phytoplankton, zooplankton, and fish, respectively, expressed in g/m^3^; *O* is the DO concentration in mg/L; $r_{P},K,g_{Z},g_{F},h_{P},h_{Z},e_{Z},e_{F},m_{P},m_{Z},m_{F}$ are growth, trophic interaction, and mortality parameters, $a_{P},b_{R},k_{2}$ are parameters of the oxygen balance, $O_{sat}\left(T\right)=O_{sat\_base}\left(1-0.02(T-20)\right)$ is the DO saturation level of the water, which depends on its temperature *T*, and $O_{sat\_base}$ is the DO saturation level at 20 °C. Descriptions, typical and normal values of growth, trophic interaction, and mortality parameters mentioned above are shown in detail in Table 3, Table 4, Table 5 and Table 6. The values and ranges shown in those tables are based on published experimental and theoretical studies in aquaculture, limnology, and ecological modeling, including classical predator–prey dynamics, functional responses, and oxygen dynamics in fish ponds [27,28,29]. Those sources provide reliable typical values and normal ranges for model parameters.

In state-space form, model (1) is expressed as

(2) $$\dot{X}=f\left(X\right)=\left[\begin{array}{c} r_{P}P\left(1-\frac{P}{K}\right)-g_{Z}\frac{PZ}{h_{P}+P}-m_{P}P\\ e_{Z}g_{Z}\frac{PZ}{h_{P}+P}-g_{F}\frac{ZF}{h_{Z}+Z}-m_{Z}Z\\ e_{F}g_{F}\frac{ZF}{h_{Z}+Z}-m_{F}F\\ a_{P}P-b_{R}\left(P+Z+F\right)+k_{2}\left(O_{sat}-O\right) \end{array}\right]$$

where $X={\left[P\;Z\;F\;O\right]}^{T}$ is the system’s state vector. This model, called the P–Z–F–O model, reflects key trophic and biochemical processes in the pond and allows prediction of its state while being computationally efficient for implementation in real-time systems. One can see from (2) that the pond ecosystem model is non-linear. At that, usually only *O* (DO concentration) is measured, and thus the measurement vector is Z1=h(X)=O . Values of state variables *P*, *Z*, and *F* usually cannot be measured directly and have to be estimated. To address that problem, the application of Extended Kalman filtering [30] is considered.

Hereinafter, notation $x_{n|m}$ represents the estimate of *x* at time *n* given observations up to and including at time m≤n , and $y_{n}$ represents the estimate of *y* at time *n*. In the EKF, for each time-step *k*, first a priori system state ${\hat{X}}_{k|k-1}$ and covariance $P_{k|k-1}$ are predicted using formulae

(3) $$\begin{array}{c} {\hat{X}}_{k|k-1}=f\left({\hat{X}}_{k-1|k-1}\right), \end{array}$$

(4) $$\begin{array}{c} P_{k|k-1}=F1_{k}P_{k-1|k-1}F1_{k}^{T}+Q_{k}, \end{array}$$

where $F1_{k}={\left.\frac{\partial f}{\partial X}\right|}_{{\hat{X}}_{k|k},u_{k}}$ is the state transition model, applied to the previous state of the system, f(X) is the function that describes the right-hand side of (2), *Q* is the covariance of the process noise, and *P* is the covariance matrix of the system. Then, innovation $Y_{k}$ , innovation covariance $S_{k}$ , and optimal Kalman gain $K1_{k}$ are calculated, and a posteriori state ${\hat{X}}_{k|k}$ and covariance matrix $P_{k|k}$ are estimated based on measured data as follows

(5) $$\begin{array}{c} Y_{k}=Z1_{k}-HX_{k-1}, \end{array}$$

(6) $$\begin{array}{c} S_{k}=H_{k}P_{k|k-1}H_{k}^{T}+R_{k}, \end{array}$$

(7) $$\begin{array}{c} K1_{k}=P_{k|k-1}H_{k}^{T}{S_{k}}^{-1}, \end{array}$$

(8) $$\begin{array}{c} {\hat{X}}_{k|k}={\hat{X}}_{k|k-1}+K1_{k}Y_{k}, \end{array}$$

(9) $$\begin{array}{c} P_{k|k}=\left(I-K1_{k}H_{k}\right)P_{k|k-1}, \end{array}$$

where *I* is the unit matrix, $H_{k}={\left.\frac{\partial h}{\partial X}\right|}_{{\hat{X}}_{k|k}}={\left[0\;0\;0\;1\right]}^{T}$ is the observation model at time *k*, and $R_{k}$ is the covariance of the measurement noise at the same step.

In order to provide biological and physical correctness of parameter estimation, the following constraints are set: $P\geq 0,\;Z\geq 0,\;F\geq 0,\;O_{min}\leq O\leq O_{max}$ , where $O_{min}$ and $O_{max}$ are the minimal and maximal DO concentrations possible. Such constraints are standard for ecological models of water ecosystems and prevent incorrect state estimation in the process of Kalman filtering. After each EKF correction step, system state estimates are projected into the domain of valid values in order to ensure that above constraints are met.

Indirect parameters (water clarity by Secchi *S*, chlorophyll concentration $C_{chl}$ ) are used for:

- initialization of initial state $X_{0}$ ;
- checking of *P* and *Z* estimation adequacy;
- correction of model parameters and matrix of noise process *Q*.

The model that empirically connects indirect parameters with phytoplankton biomass has the form

(10) $$S=c_{S}P+\epsilon_{S},\;C_{chl}=k_{chl}P$$

where $c_{S}$ and $k_{chl}$ are empiric coefficients that depend on pond morphometry (depth, area, volume, etc.); $\epsilon_{S}$ is noise or measurement error that models random factors. Zooplankton with biomass *Z* consumes phytoplankton, therefore *P* concentration will decrease with the increase in *Z*. After the introduction of coefficient α that characterizes how much phytoplankton is consumed by a unit biomass of zooplankton, Equation (10) takes the form:

(11) $$\begin{array}{c} S=c_{S}P_{eff}+\epsilon_{S}=c_{S}\left(P-\alpha Z\right)+\epsilon_{S},\;C_{chl}=k_{chl}P_{eff}=k_{chl}\left(P-\alpha Z\right) \end{array}$$

where $P_{eff}$ is the “effective” phytoplankton biomass, which is “visible” through water clarity and chlorophyll. If *Z* is small, the model reduces to the classic one: $P\approx P_{eff}$ .

The model presented above enables the estimation of *Z* through an inverse solution: given that values of key parameters—*S* and $C_{chl}$ —are known, one can calculate the proportion of phytoplankton biomass consumed by zooplankton. The indirect parameters are measured at different frequencies: key parameters are sampled 1–3 times per day, while other parameters, that may be needed for model calibration, are typically gathered only once per vegetation season. While those low-frequency measurements are not included in the measurement vector during the normal operation of the EKF, they serve a critical methodological role: they are essential for constraining the model during initialization and for the periodic refinement of its parameters in dedicated calibration phases, thereby ensuring long-term prognostic fidelity.

Use of the proposed approach to pond ecosystem modeling allows one to estimate fish pond parameters using a minimal set of sensors. Moreover, as $P_{k|k}$ and $K1_{k}$ are updated at each step of Kalman filtering, the pond model adapts to changes in the pond using measured data $Z1_{k}$ .

In a formal sense, a non-linear state-space model (2) is partially observable, as only one state variable of the vector *X* (DO concentration *O*) is measured directly. However, practical system observability is ensured by the structure of the ecological model and the presence of a strong functional relationship between phytoplankton biomass and the oxygen balance of the pond.

To analyze local observability of the non-linear system, an approach, based on Lie derivatives, is used. The Lie derivative matrix is defined as

(12) $$𝒪\left(X\right)=\left[\begin{array}{c} \nabla h(X)\\ \nabla L_{f}h\left(X\right)\\ \nabla L_{f}^{2}h\left(X\right)\\ \vdots \end{array}\right]$$

where $L_{f}h\left(X\right)$ is the Lie derivative of the observability function along the vector field f(X) . The zeroth Lie derivative has the form

(13) $$L_{f}^{0}h\left(X\right)=h\left(X\right)=O.$$

The first Lie derivative is determined by the oxygen balance dynamics

(14) $$L_{f}h=\dot{O}=a_{P}P-b_{R}\left(P+Z+F\right)-k_{2}\left(O-O_{sat}\right),$$

and its gradient by the state vector is

(15) $$\nabla L_{f}h={\left[\left(a_{P}-b_{R}\right)\;-b_{R}\;-b_{R}\;-k_{2}\right]}^{T}.$$

The second Lie derivative has the form

(16) $$L_{f}^{2}h=\frac{d}{dt}\left(\dot{O}\right)=a_{P}\dot{P}-b_{R}\left(\dot{P}+\dot{Z}+\dot{F}\right)-k_{2}\dot{O},$$

that contains non-linear combinations of variables *P*, *Z*, *F* via their own dynamics equations. For typical modes of pond operation, vectors ∇h(X) , $\nabla L_{f}h\left(X\right)$ , $\nabla L_{f}^{2}h\left(X\right)$ are linearly independent relative to variables *P*, *Z*, *O*, and as a result, the rank of the observability matrix O(X) is not lower than 3 (locally and under non-equilibrium modes of system functioning). That allows one to restore states of phytoplankton and zooplankton biomass (*P* and *Z*) from time series of DO measurements *O*.

As for fish biomass *F*, it has an indirect influence on the oxygen balance—via breathing and trophic interactions of higher order—and manifests itself mostly in Lie derivatives of second or higher order. Thus, *F* is structurally observable, but with a lower sensitivity compared to other state variables, i.e., it’s weakly observable. As a result, instant estimates of fish biomass has higher uncertainty compared to other model parameters estimates, but at the same time, long trends of *F* dynamics can be restored reliably.

One should note, that the P–Z–F–O model (2) is introduced as a generalized state-space representation of dissolved oxygen dynamics. Phytoplankton and zooplankton are included as latent variables describing internal oxygen sources and sinks rather than as management targets. For practical fish farming scenarios, the model can be reduced to a simplified oxygen–fish interaction without altering the monitoring, filtering, or forecasting algorithms.

Identifiability of model parameters using only one sensor is limited; therefore, biological parameters $r_{P}$ , $g_{Z}$ , $g_{F}$ , $m_{P}$ , $m_{Z}$ , $m_{F}$ are set a priori using one-time field measurements. At that, EKF is used mostly in order to estimate ecosystem state and not for full online parameter identification.

As for *Q* and *R*, their initial values are determined as follows. The initial value of the measurement noise co-variation *R* is defined by characteristics of the sensors, and typical values of mean square error for optical DO sensors are 0.05–0.1 mg/L, so $R_{0}=0.1^{2}=0.01$ . The process noise matrix *Q* models the system’s uncertainty and is estimated based on the expected speed of state variable change during one period of sampling Δt . The initial value of *Q* is a diagonal matrix with elements

(17) $$q_{i,i}={\left(\alpha_{i}\cdot X_{imax}\cdot \Delta t\right)}^{2},$$

where $\alpha_{i}$ is a dimensionless uncertainty coefficient, usually 0.05–0.15, and $X_{imax}$ is the maximum expected value of $X_{i}$ . In the process of filtering, *Q* and *R* are adaptively corrected based on the statistics of innovation $Y_{k}$ , defined by (5). That allows the filter to adapt to season and weather changes and to compensate for ecosystem model uncertainty and sensor noise.

Thus, the proposed approach provides practically sufficient observability for monitoring tasks, early detection of critical hydrochemical states, and decision support in pond management. Even the system with one DO sensor, integrated with the dynamic pond model via EKF, can be used as a base for pond monitoring.

The study of the pond model was divided into two stages. Stage 1, presented in this article, is aimed at validation of the algorithm on a minimal system configuration. The goal of the stage is to prove that ecosystem state may be reconstructed using just one integral parameter using model (1). That allows one to validate the core of the pond system modeling algorithm using minimal configuration, focus on the solution of the partial observability problem, and separate errors related to observability from ones introduced by additional sub-models. This approach also ensures reproducibility of the study and creates a clear benchmark for further studies.

The proposed system prototype is designed to measure DO, pH, and water temperature. That allows one to use a more sophisticated model, which takes all three hydrochemical parameters into account, thus integrating basic biological, physical, and chemical processes in the pond. Stage 2 of the study is to be devoted to the study of the complex model. The authors performed the preliminary study of the complex pond model, and it was determined that:

- A complex model is the system of 6 non-linear differential equations and has more than 30 parameters, compared to 13 parameters of (1).
- Most of the mentioned parameters depend on water temperature *T* according to the Q10 Van’t Hoff rule, and some of parameters depend on two or three variables.
- The complex model is fully locally observable, and the rank of its observability matrix is 6. Therefore, dynamics of all three biomasses *P*, *Z*, and *F* can be restored.
- Estimated computational complexity (see Appendix B) for the basic model with the DO sensor only is approximately 400 floating point operations per second (FLOPS) and for the complex model is 1350 FLOPS.

Therefore, the implementation of Stage 2, which involves the use of a significantly more complex three-sensor model, raises two fundamental scientific and technical challenges: the calibration of over 30 additional model parameters and overcoming the significant increase in computational complexity. A comprehensive solution to these problems requires dedicated experimental procedures and the development of novel algorithmic approaches, which fall outside of the scope of the present study. Consequently, a complete three-sensor model constitutes a separate, subsequent investigation, logically extending the results obtained from the minimal configuration.

During modeling, anomalies in the system state are detected:

- If any of the state variables violates physical limitations (e.g., F<0 or *O* > 20 mg/L, which is impossible).
- If the Mahalanobis distance $D^{2}=Y_{k}^{T}S_{k}^{-1}Y_{k}$ of the measured data prognosis is higher than the critical $\chi^{2}$ level (for 90% probability and number of degrees of freedom, equal to length of $Y_{k}$ ).

Anomalous values are ignored when detected, and the system switches to the simple prognosis mode described in Section 4.4. Modeling error is estimated using root mean square error (RMSE) of the state estimation. In numerical experiments performed in MATLAB, the true state of the system $X_{true}$ is known; therefore, RMSE is calculated as follows

(18) $$RMSE=\sqrt{\frac{1}{N}\sum_{k=1}^{N}{∥X_{true,k}-{\hat{X}}_{k}∥}^{2}}.$$

where $X_{true}$ is the true (simulated) state vector and $\hat{X}$ is the state estimate obtained using the EKF. In a real-world scenario, when the true system state is not known, estimation error is calculated using the innovation sequence $Y_{k}=Z1_{k}-H{\hat{X}}_{k|k-1}$ and its statistical properties. Typically, EKF reduces state estimation error by 35–60% depending on noise parameters.

Based on the modeling performed as described above for a time range of 20–30 days, critical conditions in the pond are detected:

- If the DO level is lower than the respiratory depression level or fish death level stated in Table 2, respiratory depression or fish kill is likely to occur.
- When phytoplankton biomass P>2.5 , algal bloom is likely to occur. The thershold is based on established eutrophication criteria in temperate freshwater systems [31,32].
- When fish biomass F<0.1 , fish are in danger.

In addition, dimensionless system stability index SI is estimated as

(19) $$SI=1-mean\left(\frac{s_{i}}{m_{i}}\right),$$

where $s_{i}$ and $m_{i}$ are standard deviations and mean values of system state parameters $X_{i},\;i=1\ldots 4$ over the ensemble of all states modeled in the given time range.

Modeling of the fish pond ecosystem was performed using MATLAB; software may be obtained from authors by request. An example of pond modeling along with results is presented in Section 5.1.

### 4.4. Data Processing and Decision Making Algorithms

**Figure 3:**
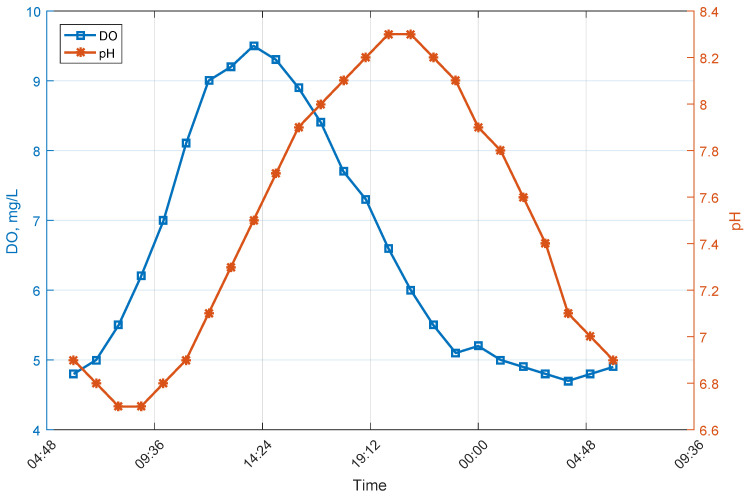
Typical DO and pH change in the fish pond during day and night.

DO and pH do not remain constant during a day. DO increases during the day and decreases at night [33]. pH level also does not remain stationary: it increases during the day up to late afternoon and then decreases at night [34]. Typical change in DO and pH in a fish pond are shown in Figure 3.

Most current studies [35], Ref. [36], concentrate on DO and pH prediction using neural networks and a combination of neural networks and wavelets [36]. Such an approach, while being effective for prediction of the hydrochemical regime, has some drawbacks:

- in order to make a reliable prediction, one needs to use extensive datasets to use wavelets and/or train the neural network and thus needs to collect data for months (authors of [35] used in their study data collected for a year);
- use of neural network by the nature of that approach does not provide any reasoning that lies behind the prognosis.

The latter issue mentioned renders the monitoring system unreliable in the eyes of farm management, as prognosis cannot be justified and possible expenses and losses due to the prognosis error are harder to eliminate. Moreover, if hydrochemical regime parameters are measured once an hour, only 24 points collected during a day are available for analysis; for DO prognosis, 150 points were used in [35]. Thus, use of another prognosis approach is desirable.

As a classical approach, in order to predict value of the parameter and, thus, water condition, least square approximation is used. The idea of a least square approximation is to minimize the difference between the approximation function and the measured parameter data:

(20) $$S\left(a_{j}\right)=\sum_{i=1}^{n}{\left(f\left(t_{i}\right)-y_{i}\right)}^{2}\to min,$$

where $t_{i}$ , $y_{i}$ are values of measurement time and measured values of the parameter in question, respectively; *n* is the number of measured data i=1…n ; f(t) is the approximation curve, $a_{j}$ are the coefficients of that curve. One should note that the moments of time $t_{i}$ , at which measured data are registered, do not have to be equally spaced; however, in most cases, that is desirable.

Typically, approximation using (20) is performed using three curves:

(21) $$\begin{array}{c} f_{1}\left(t\right)=a_{2}t+a_{1}, \end{array}$$

(22) $$\begin{array}{c} f_{2}\left(t\right)=a_{1}e^{a_{2}t}, \end{array}$$

(23) $$\begin{array}{c} f_{3}\left(t\right)=a_{3}t^{3}+a_{2}t^{2}+a_{2}t+a_{1}, \end{array}$$

i.e., linear function, exponential one, and a cubic curve. Most processes in nature manifest either linear or exponential character, therefore curve (21) or (22) is typically used.

Approximation correctness is estimated using a correlation coefficient between the measured data and approximation curve, which is expressed in the form

(24) $$r=\frac{\frac{\sum_{i=1}^{n}\left({\hat{y}}_{i}-\bar{\hat{y}}\right)\left(y_{i}-\bar{y}\right)}{n}}{\sqrt{\frac{\sum_{i=1}^{n}{\left({\hat{y}}_{i}-\bar{\hat{y}}\right)}^{2}}{n}\cdot \frac{\sum_{i=1}^{n}{\left(y_{i}-\bar{y}\right)}^{2}}{n}}},$$

where ${\hat{y}}_{i}=f\left(t_{i}\right)$ ; $\bar{\hat{y}}$ is the mean value of the approximated data, and $\bar{y}$ is the mean value of the measured data. If the value of the correlation coefficient *r* (24) is less than 0.8…0.9, approximation results cannot be considered reliable.

In order to improve *r*, automatic detection and elimination of outliers (i.e., data, measured with errors) has to be applied to measured data before approximation. In [37], a review of outlier elimination methods is presented. Use of statistical, density-based and cluster-based outlier detection methods requires knowledge of the measured signal’s statistical characteristic, typical signal data density, or measurement data clusterization, respectively. That, in turn, requires a significant volume of data to be measured and analyzed and also requires use of a powerful MCU, preferably with a floating point unit (FPU). In the case of the distributed measurement system which monitors scalar values, distance-based outlier detection methods are the methods of choice, as these ones do not require significant computation power and large datasets.

Distance-based outlier detection methods are based on the idea that data in every measured set should not differ from a given value (or one point from another) more than by some preset value. Therefore, two approaches are commonly used:

1. the value is assumed to be an outlier if it is higher or lower than a preset threshold(s);
2. the value is assumed to be an outlier if the absolute difference between that value and adjacent values is higher than a preset threshold.

Usually, a combination of both approaches is used in practice; in the second case, the threshold may be expressed as an absolute value or as a relative one. In addition, the measured point can be considered to be an outlier if the speed of the value change is higher than a given value. For a scalar value, outlier points along the timeline may be isolated or occur in groups. If the monitored parameter is measured with low frequency, outlier values will most probably be isolated points; the only exception is the case of sensor failure. Therefore, a simple algorithm that eliminates a data point if the absolute difference between its value and adjacent values is too high is assumed to be the basis of data processing.

**Figure 4:**
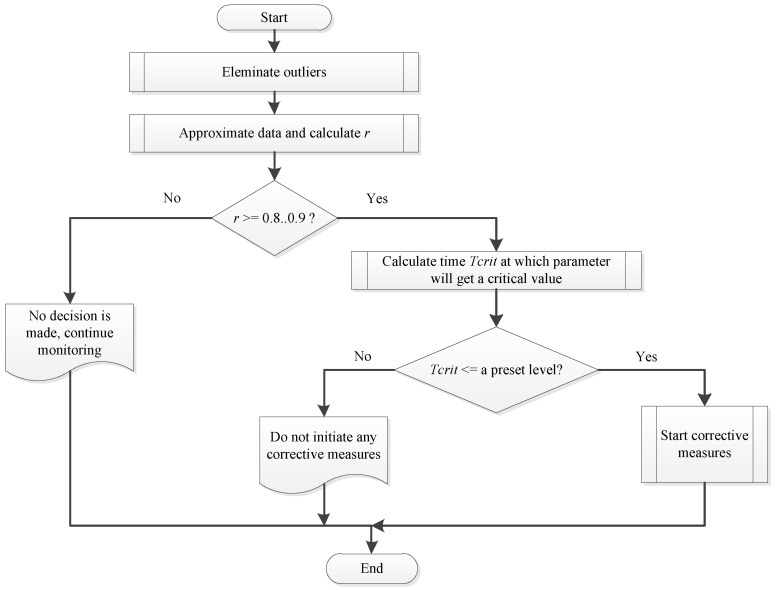
Decision-making algorithm based on the predicted value of the water parameter.

When data approximation is performed and assumed reliable, the time when the DO level will reach corresponding limiting values is presented in Table 2, and the time when pH value will become higher than 8.5 for carp fish, higher than 8.0 for salmon and trout, or lower than 6.5 is calculated. The ratio of the pH level near the water surface and near the pond bottom is also taken into account (it should not be close to 1.05 [1]). If temperature stratification is detected, then water in the pond is either mixed or discharged near the pond bottom, and aeration is applied. If any condition that leads to fish kill is met within a predefined time interval, respective corrective measures are also initiated. The decision-making algorithm for any water parameter value is shown in Figure 4.

Pseudo code of the algorithm presented above and implemented as the MakeDecision procedure is as follows (Algorithm 1):
**Algorithm 1** Decision-making algorithm based on the predicted value of the water parameter
 1:**procedure** MakeDecision(Measured data, curve type, critical value) 2:         ▹Measured data are usually given as an array of points $\left(t_{i},y_{i}\right),\ldots \left(t_{n},y_{n}\right)$ , curve type is one of those defined by (21)–(23), critical value for each water parameter is set according to reasoning presented above in the Section 3 3:      Eliminate outliers in measured data 4:      Approximate measured data with the selected curve 5:      
$r\leftarrow \frac{\frac{\sum_{i=1}^{n}\left({\hat{y}}_{i}-\bar{\hat{y}}\right)\left(y_{i}-\bar{y}\right)}{n}}{\sqrt{\frac{\sum_{i=1}^{n}{\left({\hat{y}}_{i}-\bar{\hat{y}}\right)}^{2}}{n}\cdot \frac{\sum_{i=1}^{n}{\left(y_{i}-\bar{y}\right)}^{2}}{n}}}$
 6:      **if** r≥0.8…0.9 **then** 7:           Calculate time Tcrit at which parameter will get critical value 8:           **if** Tcrit < preset level **then** 9:                 Start corrective measures10:               **return**11:         **else**12:               Do not initiate corrective measures13:               **return**14:         **end if**15:    **else**16:         No reliable data - do not initiate corrective measures17:         **return**18:    **end if**19:**end procedure**


Preset levels of time intervals for taking corrective measures should be established at the level of 3–5 h before a predicted problem and corrected in practice. Corrective measures are usually finished when water condition is normalized.

Using approximation-based prediction, one has to note the following:

- After any event, that may severely change condition of water in the pond, i.e., after treating the pond with chemicals, introduction of fertilizers, aeration, very heavy rain, moving fish from one pond to another, etc., all data measured on the pond before the event should not be used for prediction of hydrochemical regime parameters. In order to obtain a correct prognosis, one has to use data collected after the mentioned event.
- Care must be taken when analyzing and comparing data, collected in different ponds—especially when those ponds are from different farms. Even if parameter values are similar, that does not mean that parameter will behave similarly in both cases; e.g., different water supplies may significantly alter water parameter dynamics. The same is true for data collected in one pond but during different seasons (in summer and in winter, in spring and in summer, etc.).
- If any new unusual trends manifest themselves in the data, advise of an experienced fish farming practitioner is desired, as those trends may be related to a problem that may require an additional diagnosis and elimination.

One should also note that approximation-based prediction is a tool of operative control, and as such is suitable only for a short-term prognosis (usually for a period of some hours). Therefore, in order to make a long-term prognosis (e.g., during at least 20–30 days), use of a deterministic model, presented in Section 4.3, is one of the possible solutions. Prognosis for a one-year production cycle, if needed, should be implemented on the server, as that requires vast datasets and a significant computing power to function properly.

### 4.5. Measurement Module

Each measurement module should contain:

- sensors with wires and connectors;
- waterproof case (preferably IP68);
- board with 32-bit MCU that contains analog–digital converters (ADCs), I^2^Cs, and universal asynchronous receivers/transmitters (UARTs);
- LoRa or LoRaWAN module that provides connection to LoRaWAN network;
- accumulator and/or another power source;
- power conversion unit.

The MCU and LoRa/LoRaWAN modules may be replaced with a dedicated system on a chip (SoC), like ASR6601 [38] or STM32WL55xx devices [39]. As a power source, a solar cell may be used in addition to an accumulator. The basic scheme of the measurement module, selected for implementation, along with its description, is shown in Appendix C.

The module is to be powered by Li-Ion cells and solar cells in order to improve the time of its autonomous operation. All electric parts, excepts sensors with their cables and connectors, antenna, and solar cell are to be placed inside the IP68 case. As a result, the measurement module can be installed and should successfully operate on a buoy at any point of the pond.

### 4.6. Software Description

Software of the system is to be organized in two levels:

- lower-level firmware that provides measurement, initial data analysis, basic hydrochemical regime modeling and prognosis and transmits data to a higher level using LoRaWAN;
- higher-level software that provides data collection, data storage in a database, comparative analysis, and service to clients via local network or Internet (if needed).

Lower-level firmware will be running on MCUs of measurement modules. General functions of the firmware include water parameter measurement and automated outlier elimination. The pond model with EKF and basic trend analysis should also be included in firmware. Each measurement module operates as a class A LoRaWAN device, which provides measurement data to higher-level software with a given period of time in order to save the energy of the accumulator.

Most of the time, module will sleep and will initiate measurement only at preset time moments. The measurement schedule may be changed if system prognoses fish kill (more frequent measurement), when water parameters are normalized (less frequent measurement), or by user’s request. As stated above, basic data processing, including outlier detection and prognosis of water parameters in the measurement point, will be performed by the module in order to reduce data flow. In order to decrease influence of random noise, data measured at each moment of time in the schedule will be averaged during measurement.

General algorithm of the measurement module’s firmware operation is as follows (Algorithm 2):
**Algorithm 2** General algorithm of the measurement module’s firmware operation
 1:Power on module/Get it out of deep sleep mode 2:Read configuration (including curve type, critical value) from flash memory 3:Initialize sensors 4:Read data from sensors, average them 5:Turn sensors off 6:Use EKF (5)–(9) on measured data 7:Add corrected measurement results to Measured data array 8:**for** each sensor **do**                     ▹Call routine that clears and processes measured data 9:      MakeDecision(Measured data, curve type, critical value)10:**end for**11:Save processed measurement data into the local flash12:Send processed measurement data to higher level via LoRaWAN13:Receive configuration or commands from higher level14:**if** There are any configuration changes **then**15:     Save changes to flash memory16:**else if** Significant changes (chemical treatment, etc.) require data reset **then**17:     Reset data buffers18:**else if** Other command received **then**19:     Process that command20:**end if**21:Setup wake-up timer for the next measurement cycle22:Put module into deep sleep23:Wait for wake-up event24:Go to step 1


Such an algorithm of operation allows one to provide low power consumption and ensure that all data are processed at a low level of the system before sending them to a higher level. As for computation complexity, most of calculations can be performed using single precision arithmetic; moreover, fixed point may also be used—as 32 bit MCUs have good performance if 32 bit integer arithmetic is used. As for real-time feasibility, if data are measured once an hour according to recommendations presented above, processing can be considered real-time even if computation will take one–two minutes.

As a base for the firmware, the open-source LoRa Basic Modem LoRaWAN stack developed by Semtech [40] is to be used; its main advantage is its portability between different MCUs. In order to simplify the measurement unit, Activation By Personalization (ABP) is to be used to authenticate the unit in the LoRaWAN network. User-defined static Device Address (DevAddr), Network Session Key (NwkSKey), and Application Session Key (AppSKey) will be stored in the end-device and may be changed using an external interface both during commissioning and in the process of operation. Over The Air Activation (OTAA) may also be used in the future if necessary.

Higher-level software consists of the LoRaWAN network server that receives measured data, LoRaWAN application server with a database that collects and stores those data, and a client software that may provide long-term pond modeling, data visualization, trend analysis, prognosis, and corrective measures.

In order to exclude data leak to the Internet and prevent unauthorized access to any devices that can change pond condition (e.g., inlet and discharge), use of a local network is planned. Therefore, both LoRaWAN servers with a database should run on a dedicated server hardware, which has to operate and be available 24/7/365.

As a LoRaWAN network server, a number of software packages may be used. Some of those include:

- The Things Stack [41];
- Activity servers [42];
- Chirpstack [43];
- LORIOT [44];
- Multitech Network Server [45], and may others.

Most of those servers store data in a cloud; some of them operate in public networks with many restrictions and require payment for commercial usage. Of those servers listed above, Chirpstack is the only open-source and free LoRaWAN Network Server.

Chirpstack is targeted on Linux, but using Docker and Docker Compose it can be also be run under Windows and Mac OS. It provides both the Network Server and Application Server and can use SQLite or Postgre SQL as a database. Both installers and ready-to-use Docker images are available on Chirpstack’s official site download page. In order to connect the LoRaWAN network server to external clients, HTTP, Web Socket protocol, or MQTT may be used; in real-life scenarios, secure versions of those protocols should be used.

Client software of the monitoring system has to provide to its end-users the following features:

- trend analysis of measured water parameters;
- analysis and long-term prediction of hydrochemical regime using the model of the pond;
- prognosis of fish kill based on that analysis;
- and, optionally, initiation of corrective actions, including aeration and water exchange in the pond.

The functions mentioned above are easy to implement on any personal computer, under either Windows, Mac OS, or Linux. Mobile software, based on Android or iOS, may also provide real-time indication of water parameters and trend analysis. Any automated corrective actions (if they are possible) should be initiated by the monitoring system and acknowledged by the user (fish farm specialist or pond owner). Those actions may include water aeration, mixing water or discharge of lower layers of water in order to break stratification, etc. One should note that if the monitoring system can initiate any corrective measures, it is preferable that any actuator mechanisms which control aerators, water inlets, water discharges, and other equipment were physically inaccessible from external networks (especially from the Internet).

At last, all results of water parameters prediction that indicate possible problems (including fish kill) and all corrective actions taken should be stored in the event log, placed on the same server hardware that hosts LoRaWAN servers. To that end, the same database server that is used to store measured data may be used.

## 5. Results and Discussion

The system proposed above is now in the process of implementation; therefore, only estimation of its parameters and results of simulation are available for now. Results of that estimation and simulation are presented below.

### 5.1. Test of Fish Pond Modeling

**Figure 5:**
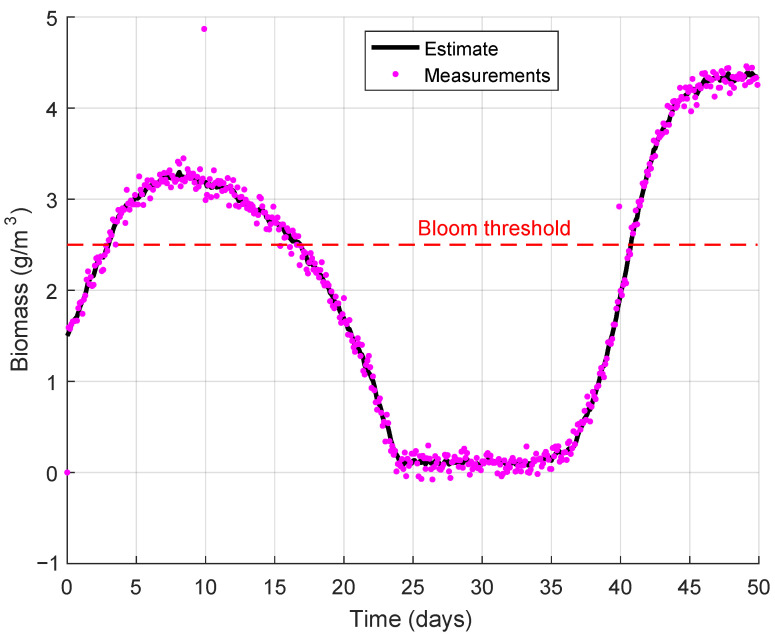
Results of phytoplankton modeling. Measured data (magenta points) and estimation results (black solid line) are in good agreement. Algae bloom is possible when phytoplankton biomass concentration is higher than the bloom threshold.

**Figure 6:**
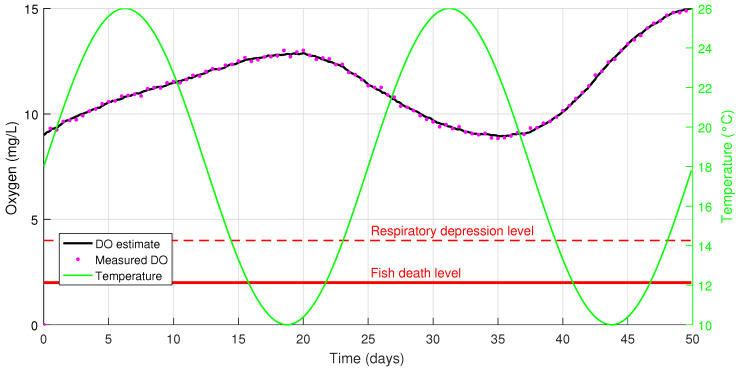
Results of DO level modeling. Measured data (magenta points) and estimation results (black solid line) are in good agreement. DO is higher than respiratory depression and fish death levels, so any problems are unlikely to occur.

First, modeling of the fish pond was performed with the following model parameters: $r_{P}$ = 0.8 1/day, K=5.0 g/m^3^, $g_{Z}=0.6$ 1/day, $g_{F}=0.3$ 1/day, $h_{P}=0.5$ g/m^3^, $h_{Z}=0.3$ g/m^3^, $e_{Z}=0.4$ , $e_{F}=0.3$ , $m_{P}=0.1$ 1/day, $m_{Z}=0.08$ 1/day, $m_{F}=0.05$ 1/day, $a_{P}=0.3$ mg/(L·day), $b_{R}=0.1$ mg/(L·day), $k_{2}=0.15$ 1/day. Base oxygen saturation was assumed to be 10 mg/L, medium temperature of the water was 18 °C, water temperature amplitude change was 8 °C, and temperature fluctuation period was set to 25 days. Measurement noise was 0.08, process noise was 0.02, and initial state vector $X_{0}={\left[1.5\;0.8\;0.3\;9.0\right]}^{T}$ . Modeling results during the period of 50 days are shown in Figure 5 and Figure 6.

As one can see from Figure 5, phytoplankton biomass becomes higher than algae bloom threshold from day 3 to day 16 and from day 41 to day 50; that may lead to algae bloom and, subsequently, to fish kill. DO level changes as shown in Figure 6; it fluctuates from 9 to 15 mg/L, while temperature oscillates from 10 to 26 °C. At that, estimated data are in good agreement with measured ones for both values due to application of the Kalman filter. Thus, the proposed model allows one to estimate DO level and other parameters of the pond state and may be used in fish pond state monitoring.

### 5.2. Preliminary Tests of System Concept and Algorithms

Preliminary tests of the system concept and algorithms were carried out in the Kyiv Reservoir (50° $38^{\prime }$ $25.5^{\prime \prime }$ N 30° $26^{\prime }$ $42.2^{\prime \prime }$ E) at a depth of 20 cm (near the surface). Model parameters were calibrated using data collected in September 2025, with chlorophyll-a (a key pigment of oxygenic photosynthesis) concentration as the input variable. Chlorophyll-a measurements were made once every three days. Chlorophyll-a concentration declined from values of 13.8 μg/L in early September to 6.5 μg/L at the end of the month.

DO and temperature measurement were performed using an Atlas Scientific Gen 3 Industrial D.O. Probe sensor [22] with an EZO^TM^ auxiliary board. The WeAct Black Pill board with a STM32F401CEU6 microcontroller was used for sensor control, pre-processing, and data storage. The sensor was calibrated according to the manufacturer’s recommendations. The system was programmed to measure the concentration of DO (mg/L $O_{2}$ ) and water temperature at 1 h intervals, and DO, water temperature, and measurement time were collected and stored. Collected data were saved in .csv format. Data processing was performed in the MATLAB R2016b environment. The processing main pipeline included:

1. Import and validation: Loading the .csv file with the **readtable** function (DateTime text column is converted to **datetime** format).
2. Outlier elimination: detection and interpolation of anomalous values using the median filter(**movmedian** function).
3. Aggregation: calculation of daily statistics (minimum, maximum values) for further analysis.

**Figure 7:**
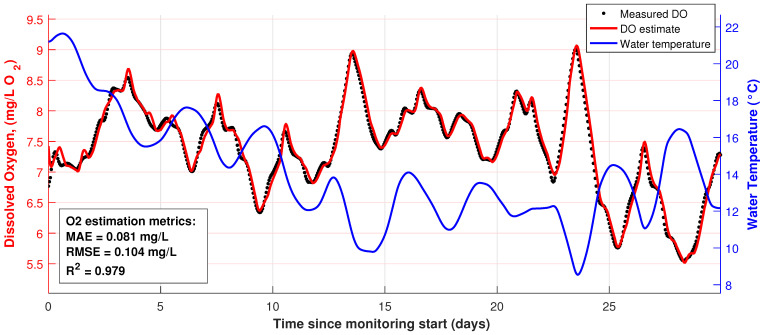
Time series of DO dynamics and water temperature from the field tests (50° $38^{\prime }$ $25.5^{\prime \prime }$ N 30° $26^{\prime }$ $42.2^{\prime \prime }$ E, at 20 cm depth, September 1 to September 30, 2025).

In Figure 7, DO and water temperature are plotted. Data were recorded from September 1 to September 30, 2025. The black dots represent the measured DO concentrations. The red solid line shows the DO predicted in real time by the Extended Kalman Filter (EKF). The blue line indicates the measured water temperature, a key driver of DO dynamics.

EKF effectiveness was assessed using comparison of predicted DO values and empirical (measured) data. The system demonstrated high accuracy with RMSE=0.104 mg/L and determination coefficient $R^{2}=0.979$ . The mean absolute error of DO prognosis is 0.081 mg/L. Those results clearly show that the model reflects dynamics of the pond’s oxygen regime adequately and thus can be used in the operative monitoring.

**Figure 8:**
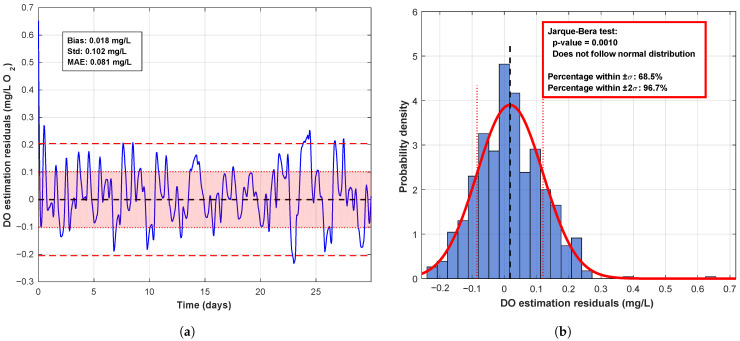
Analysis of DO prediction residuals: (**a**) Time series of residuals (measured DO—EKF-predicted DO). (**b**) Histogram of the residuals with an overlaid normal distribution curve. Key metrics: Bias (mean) = 0.018 mg/L, Standard Deviation = 0.102 mg/L.

A detailed statistical assessment of the prediction residuals (differences of estimated and measured DO values) is presented in Figure 8.

The residuals exhibit a negligible systematic bias of 0.018 mg/L and a standard deviation of 0.102 mg/L, yielding a mean absolute error (MAE) of 0.081 mg/L. The apparent contradiction between the Jarque–Bera test result and the empirical distribution is resolved by distinguishing statistical from practical significance: while the test’s high sensitivity indicates a formal deviation from perfect normality (*p* = 0.0010), the actual error distribution is practically normal. Key evidence of this is the proportion of residuals within ±1σ and ±2σ of the mean, which are 68.5% and 96.7%, respectively. These empirical quantiles show close agreement with the theoretical expectations for a Gaussian distribution (68.3% and 95.5%). Therefore, practical insignificance of these deviations, combined with the known robustness of the Kalman filter to minor violations of normality, validates its applicability and ensures high forecasting accuracy within this operational framework.

As one can see from the results of preliminary tests, a system built under the presented concept can clearly be implemented. At that, the use of EKF allows one to estimate hydrochemical pond parameters—at least DO—with RMSE=0.104 mg/L. Thus, the system that uses the Atlas Scientific Gen 3 Industrial D.O. Probe sensor can be used as a base for further system development on the next stage of this study.

### 5.3. Data Processing Simulation

**Figure 9:**
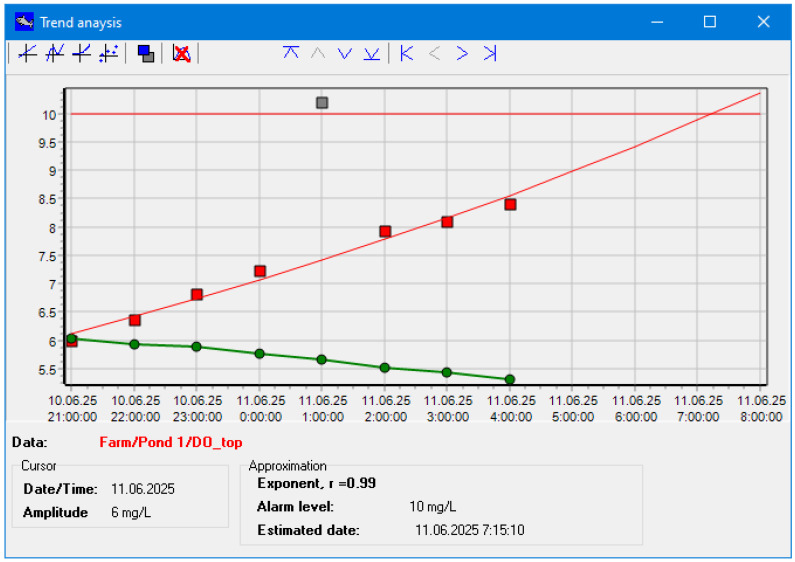
Trend analysis window of the client software, showing DO (red squares) and pH (green circles) values.

The results of application of the proposed approach to approximation and outlier elimination are shown below. An example of the Windows-based software under development in trend analysis mode with generated demo data is shown in Figure 9.

As one can see from Figure 9, the DO level in the pond near its surface is increasing, and stratification seems to be forming in the pond. The critical DO level in the pond is estimated to be attained at 7:15:10. Outliers are shown in gray color and are not included into approximation.

As both DO and pH change during the day (see Figure 3), approximation must take that into account. The simplest way to ensure good approximation is to divide obtained data into two regions: first from 6 a.m. until 9 p.m. and second from 9 p.m. to 6 a.m. In such a way, one can ensure monotonic changes in DO and pH at night, and almost monotonic during a day, and that will allow one to use approximation while minimizing its errors. However, simple analysis of the absolute maximum and minimum of the water parameter during a day may also be used in order to establish mentioned regions for the next operation day; as light day length changes slowly, such an approach should be an acceptable solution.

### 5.4. Design Parameters Estimation and Discussion

Power efficiency of the design is the key to successful system deployment. Components, selected for system implementation, are shown in Table 7.

The estimated power budget of the measurement module with Atlas Scientific sensors is presented in Table 8.

As module activity time, which includes measurement, data processing, and transmission, is 1 min maximum, and measurement takes place once an hour, total power consumption per one day is 1.5332 W·h, and, therefore, a single 18,650 3.7 V Li-Ion battery with 12.95 W·h capacity will allow the module to run for 15.6 days—if no solar cell is used. Use of 12 such batteries will allow the module to operate for half of a year (188 days) on a single charge. Use of a solar cell allows for charging of the battery during a day, thus the number of Li-Ion batteries needed per module can be reduced.

As for the ease of the monitoring system’s implementation, it is based mostly on the standard off-the-shelf components, and its hardware requires a little development time to implement. In order to implement such a system, one has to put the most effort into development and manufacturing of the measurement module with its firmware and client software, as most of the high-level software is standard.

Next thing to consider is the system’s accuracy. As sensors proposed for use are digital, system accuracy is determined by the errors of sensors themselves, as sensing elements, amplifiers, signal conditioners, and ADCs are contained inside those sensors. The measurement module is also digital and, therefore, does not introduce an additional error in digital input signal. The only source of errors, that may have an impact on the measured values, is the power source; as power is provided by an accumulator, any power fluctuations are unlikely to occur. With Atlas Scientific sensors, measurement errors are ±0.05 mg/L for DO, ±0.002 for pH, and up to ±0.2 °C for temperature. That is more than enough for fish farming.

Next is the system’s scalability. Theoretically, LoRaWAN allows one to use up to 10,000 nodes (modules); however, to that end, one has to correctly plan both network and data rate. As for network planning, use of a single 8-channel gateway usually allows one to receive data from 32 to 128 modules. At that, use of a high spreading factor (i.e., SF10–SF12) allows one to achieve the longest range between nodes but also limits the size of the LoRa packet payload to 51 bytes and increases time on air for 16 bytes payload up to 1646.6 ms (868 MHz frequency band, bandwidth 125 MHz). For spreading factor SF7, the maximum payload size is 222 bytes, and time on air is 66.8 ms with the same channel characteristics. Moreover, in order to obtain distance of 10–15 km between modules in rural areas, one has to place module and gateway antennas in a line of sight. So, in each case of monitoring system deployment, the network has to be set up individually, taking the position of modules and gateway antennas into account. In cases of very large areas or when 100+ modules are to be used, use of many gateways that transfer data to the LoRaWAN network server is desired in order to speed up data transfer and simplify system adjustment. However, such a solution will increase the system’s overall cost.

The last, but not the least, is the system’s cost. Overall cost depends on the number of modules used, and the most expensive parts of the measurement module are its sensors. If Atlas Scientific sensor’s retail price lies in the range of $100–$300 (as of 2025, depending on the lot size), industrial-grade sensors of other manufacturers are much more expensive (usually $1000+). However, if the loss due to one fish kill is estimated to be tens of thousands dollars, use of the monitoring system can clearly be justified.

## 6. Conclusions

This study presents a conceptual design of a hydrochemical monitoring system tailored for large-scale aquaculture, contributing to the development of smart Agriculture 4.0 solutions. The proposed system architecture, combining low-power sensor nodes, LoRaWAN communication, and hierarchical data processing, establishes a foundation for developing an operational monitoring platform. The core of the approach is integration of a dynamic pond ecosystem model with an Extended Kalman Filter (EKF). It has been demonstrated that even a minimal configuration using only a DO sensor allows for the estimation of pond hydrochemical state, thereby validating the fundamental feasibility of state reconstruction under conditions of partial observability. Such an approach also allows one to explain and justify the reasoning behind every recommended corrective action. A basic set of sensors (DO, pH, temperature) provides a suitable basis for measurements, with the architecture allowing for the integration of other sensor types. It is important to note that the current work concludes the first stage of research, corresponding to Technology Readiness Level (TRL) 3—experimental proof of concept.

The next stage of research (targeting TRL 4–5) will focus on:

1. Calibration and validation of the basic model using data from a specific fish farm.
2. Establishment of operational thresholds for key water quality parameters (DO, pH, T) with the involvement of experienced aquaculture practitioners.
3. Field testing and optimization of the hardware to ensure durability and energy efficiency under real-world conditions.

In the next research stage, further system development is planned, including implementation of an expanded model that accounts for pH and temperature dynamics. This step will require overcoming specific scientific and technical challenges, including the calibration of over 30 additional parameters and optimization of computation. Within this stage, a pilot deployment, final system tuning, and the development of practical recommendations for fish farms management are planned.

## Figures and Tables

**Table 1:** Comparison of features of aquaculture monitoring systems.

Publication	Spatial Scale	Sensors Used	Communication	Modeling and Decision Support
Chatziantoniou et al. [10]	Scattered marine areas	DO, temperature, satellite imagery	Internet (wired and Wi-Fi)	Bioenergetic model of fish growth
Akhter et al. [11]	Small fisheries	DO, temperature, pH, nitrate, phosphate, calcium, magnesium	LoRa	Not specified
Khudoyberdiev et al. [12]	Tank aquaculture	Temperature, pH, and water and conductivity levels	Not specified	Neural Network + Fuzzy logic
Al-Mutairi and Al-Aubidy [13]	Small fisheries	DO, pH, turbidity, temperature, electric conductivity, total dissolved solids, water level	Wi-Fi	Fuzzy logic
Mohd Jais et al. [14]	Tank aquaculture	Temperature, DO, pH, ammonia, and salinity	Wi-Fi	None (to be implemented)
Mat Tahir et al. [15]	Tank aquaculture	pH, food level	Wi-Fi	None
Tolentino et al. [16]	Intensive (tank) aquaculture	Temperature, DO, pH, oxidation–reduction potential, turbidity, salinity	LoRaWAN	Not specified
Chen et al. [17]	Small fisheries, tank aquaculture	Temperature, pH, DO, water level	LoRaWAN	None (to be implemented)
Tamim et al. [18]	Not specified	pH, temperature (DO, ammonia measured offline)	Wi-Fi	None
Salem et al. [19]	Tanks (wastewater plant)	pH and temperature	Wi-Fi + cellular networks	None
Valarmathi et al. in [20]	Tanks (wastewater plant)	Turbidity, total dissolved solids, temperature, and humidity	Wi-Fi	None
Chavan et al. [21]	Tanks (wastewater plant)	pH, DO, electrical conductivity, TDS, turbidity, and temperature	Wi-Fi + cellular networks	Machine learning (method unspecified)

**Table 2:** DO levels at which respiratory depression and fish death occur at 0–0.5 °C (Data from [25]).

Fish Species	Respiratory Depression Level, mg/L	Fish Death Level, mg/L
Nelma	7.5–6.0	4.5–4.0
Sterlet	7.5–6.0	3.5
Muksun	4.5–3.0	2.0–1.5
Peled	4.5–3.0	1.5–1.0
Common dace	4.5–3.0	1.2–0.8
European perch	4.5–3.0	1.1–0.6
Ide	4.5–3.0	0.5
Roach	3.0–2.0	0.7
Northern pike	3.0–2.0	0.6–0.3
Crucian carp	2.0–1.0	0.1

**Table 3:** Phytoplankton parameters (data from [27,28,29]).

Parameter	Designation	Measurement Units	Typical Value	Range of Normal Values
Growth rate	$r_{P}$	day^−1^	0.8–1.2	0.5–2.0
Carrying capacity	*K*	g/m^3^	5.0	3.0–8.0
Half-saturation constant	$h_{P}$	g/m^3^	0.5	0.1–1.0
Mortality rate	$m_{P}$	day^−1^	0.1	0.05–0.2

**Table 4:** Zooplankton parameters (data from [27,28,29]).

Parameter	Designation	Measurement Units	Typical Value	Range of Normal Values
Grazing rate	$g_{Z}$	day^−1^	0.6	0.3–1.0
Assimilation efficiency	$e_{Z}$	dimensionless	0.4	0.3–0.6
Half-saturation constant	$h_{Z}$	g/m^3^	0.3	0.1–0.5
Mortality rate	$m_{Z}$	day^−1^	0.08	0.05–0.15

**Table 5:** Fish parameters (data from [27,28,29]).

Parameter	Designation	Measurement Units	Typical Value	Range of Normal Values
Predation rate	$g_{F}$	day^−1^	0.3	0.1–0.5
Assimilation efficiency	$e_{F}$	dimensionless	0.3	0.2–0.4
Mortality rate	$m_{F}$	day^−1^	0.05	0.02–0.1

**Table 6:** Oxygen balance parameters (data from [27,28,29]).

Parameter	Designation	Measurement Units	Typical Value	Range of Normal Values
Oxygen production	$a_{P}$	mg/L/day	0.3	0.1–0.5
Oxygen consumption	$b_{R}$	mg/L/day	0.1	0.05–0.2
Reaeration coefficient	$k_{2}$	day^−1^	0.15	0.1–0.3
Base oxygen saturation (at 20 °C)	$O_{sat\_base}$	mg/L	9.1	8.0–10.0

**Table 7:** Components selected for system implementation.

Component	Model
DO sensor	Atlas Scientific Gen 3 Industrial D.O. Probe with EZO^TM^ board
pH sensor	Atlas Scientific Gen 3 Mini Lab Grade pH Probe with EZO^TM^ board
MCU board	WeAct Black Pill with STM32F401CEU6 MCU
LoRa Module	RFM-95W

**Table 8:** Estimated power budget of the measurement module with Atlas Scientific sensors.

Module Component	Operation Mode Consumption, W	Sleep Mode Consumption, W
DO sensor	0.6240	0
pH sensor	0.0915	0
MCU board	0.6600	0.005
LoRa Module	0.3960	3.3 × 10^−6^
**Total**	**1.7715**	**0.005**

**Figure A1:**
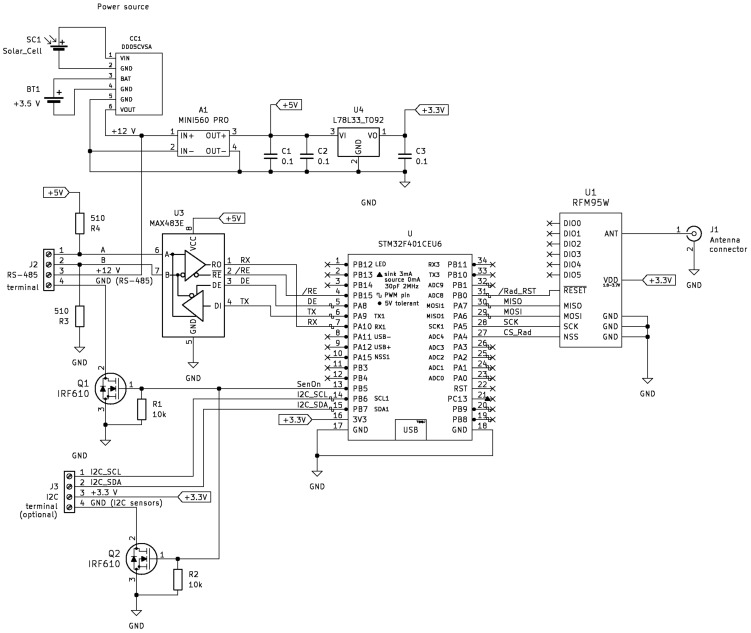
Measurement unit schematics.

## Data Availability

Data may be obtained by request.

## Abbreviations

The following abbreviations are used in this manuscript:

ABP	Activation By Personalization
ADC	Analog–Digital Converter
AppSKey	Application Session Key
ARM	Advanced RISC Machine
DevAddr	Device Address
DO	Dissolved Oxygen
EKF	Extended Kalman Filter
FLOPS	Floating Point Operation Per Second
FPU	Floating Point Unit
I^2^C	Inter-Integrated Circuit
IoT	Internet of Things
LoRa	Long Range
LoRaWAN	Long-Range Wide-Area Network
MAE	Mean Absolute Error
MCU	Microcontroller Unit
MQTT	Message Queue Telemetry Transport
NwkSKey	Network Session Key
OTAA	Over The Air Activation
RMSE	Root Mean Square Error
RTU	Remote Terminal Unit
SoC	System on a Chip
SPI	Serial Peripheral Interface
TDS	Total Dissolved Solids
TRL	Technology Readiness Level
UART	Universal Asynchronous Receiver/Transmitter
UAV	Unmanned Air Vehicle
Wi-Fi	Wireless Fidelity

## Appendix A. Data of Commercially Available Industrial-Grade and Laboratory-Grade DO and pH Sensors

Characteristics of some of the industrial-grade and laboratory-grade sensors available on the market that can be used for hydrochemical regime monitoring in a fish pond are shown in Table A1 and Table A2. As for the temperature sensor, it is present in either DO or pH sensors.

**Table A1:** Characteristics of DO sensors.

Parameter	Atlas Scientific Gen 3 Industrial D.O. Probe [22]	BOQU IOT-485-DO [46]	AQUALABO OPTOD [47]	YOKOGAWA DO71 [48]
Measurement range, mg/L	0–100	0–20	0–20	0–22.5
Accuracy, mg/L	±0.05	±0.20	±0.10	±0.05
Temperature range, °C	1–99	0–65	0–50	0–50
Built-in temperature sensor	Yes	Yes	Yes	Yes
Temperature accuracy ^1^, °C	±(0.15+(0.002t)	±0.5	±0.5	≤1.0
Maximum depth, m	212	-	50	120
Output interface	Analog (galvanic) ^2^	RS-485 Modbus RTU	RS-485 Modbus RTU and SDI-12	RS-485 Modbus RTU
Time before recalibration	1 year	-	1 year	1 year
Maintenance period	1–2 years	-	2 years	1 year

^1^*t* is the measured temperature value expressed in °C. ^2^ May be converted to Inter-Integrated Circuit (I^2^C) interface using an external EZO^TM^ board.

**Table A2:** Characteristics of pH sensors.

Parameter	Atlas Scientific Gen 3 Mini Lab Grade pH Probe [49]	BOQU BH-485-PH [50]	AQUALABO PHEHT Monobloc [47]	YOKOGAWA PM21 with FLXA21 [51]
Measurement range	0–14	0–14	0–14	0–14
Accuracy	±0.002	±0.1	±0.1	±0.01
Temperature range, °C	−5–99	0–65	0–50	0–60
Built-in temperature sensor	No	Yes	Yes	Yes
Temperature accuracy, °C	-	±0.1	±0.5	±0.3
Maximum depth, m	78	-	50	50
Output interface	Analog (voltage) ^1^	RS-485 Modbus RTU	RS-485 Modbus RTU and SDI-12	HART, Profibus, Fieldbus ^2^
Time before recalibration	1 year	> 60 days	1 year	-
Maintenance period	1 year	-	1 year	-

^1^ May be converted to I^2^C interface using an external EZO^TM^ board; ^2^ May be converted to Modbus RTU using YOKOGAWA FLXA402 device.

As one can see from the tables presented above, most of the industrial-grade sensors have a Modbus RTU interface. The use of a digital interface eliminates the need for measurement channel re-calibration when replacing a faulty sensor. Analog sensors proposed by Atlas Scientific may be converted to the I^2^C interface, using either the Atlas Scientific (EZO^TM^) interface board or a custom-made board in the measurement module. The main drawback of the digital output is that the Modbus register map is unique for each sensor type. As a result, sensors of different manufacturers are not interchangeable.

As for other parameters, all sensors are rather close in terms of measurement range and operating temperature range and have comparable measurement precision. A maximum immersion depth of 50 m and more is redundant for most fish farming applications. So, the limiting factors of sensor choice are maintenance period and price.

Many DO sensors nowadays are optical rather than electrochemical; therefore, no frequent electrode change and re-calibration are needed. The same is true for temperature sensors, which mostly use resistive temperature detectors. pH sensors, however, in most cases still require use of gel and electrodes, and, therefore, sensor maintenance time and lifetime are defined mostly by service life of electrodes. So, when selecting the pH sensor, one has to take both price and life of a sensing electrode into account.

Sensor maintenance may include not only electrode or gel replacement. Periodic cleaning of membranes or optical windows may also be necessary if sensor operating conditions are harsh. To that end, most manufacturers propose cleaning systems, which allow one to clear sensors in place of operation, thus decreasing maintenance time. However, use of such a system increases total cost and energy consumption of the measurement module.

If identifying and measuring of contaminants in water is necessary, usually, offline measurement using additional sensors is performed. General review of sensors for detection and monitoring of contaminants in wastewater is presented in [52] and may be used as a base for selection of such sensors. Remote sensing technology and sensors for water quality monitoring are also discussed in [53].

## Appendix B. Estimation of Computation Complexity for Pond Models

Computational complexity of EKF is determined by the dimension of the tasks (number of state variables $n_{x}$ and measurements $n_{z}$ ) and the number of matrix operations needed at each step. This analysis is performed for two stages of conceptual development of the monitoring system with model (1) and with the model that takes DO, pH, and temperature into account, and analysis results are shown in Table A3.

**Table A3:** Computation complexity for pond models.

Parameter	Stage 1 (Model (1))	Stage 2 (Complex Model)
Concept	Model with partial observability	Full biogeochemical model with full observability
State dimension ( $n_{x}$ )	4 (*P*, *Z*, *F*, *O*)	6 (*P*, *Z*, *F*, *O*, pH , *T*)
Measurement dimension ( $n_{z}$ )	1 (*O*)	3 (*O*, pH , *T*)
Model integration	∼24 FLOPS (4 simple equations)	∼84 FLOPS (6 simple equations)
Jacobian $F_{k}$	∼96 FLOPS (4 × 4 matrix)	∼360 FLOPS (6 × 6 matrix))
Covariation prognosis	∼144 FLOPS (2 × $4^{3}+4^{2}$ )	∼468 FLOPS (2 × $6^{3}+6^{2}$ )
Kalman correction	∼40 FLOPS	∼392 FLOPS
**Total complexity (per one step)**	**≈304 FLOPS**	**≈1304 FLOPS**

One should note that data presented in Table A3 are approximate and reflect the number of floating point arithmetic operations (additions, subtractions, multiplications, and divisions). Model integration (Euler scheme) complexity strongly depends on the complexity of the right-hand side of the model equations. The most significant contribution to the complexity is provided by matrix operations, especially by covariance prediction (4) and Kalman correction (5)–(9).

Calculations requiring both 304 FLOPS and 1304 FLOPS complexity can be performed on 32-bit MCU with built-in FPU (e.g., STM32F401CE), especially if data are collected rarely, e.g., once an hour.

## Appendix C. Schematics of the Measurement Module

Basic schematics of the measurement module are shown in Figure A1.

As one can see from Figure A1, the measurement unit is based on the WeAct Black Pill board featuring STM32F401CEU6 MCU. As a LoRa module, RFM-95W with a serial peripheral interface (SPI) interface is used. Taking data shown in Table A1 and Table A2 into account, the module allows RS-485 and galvanic (using I^2^C interface boards) sensors to be used. In both cases, sensors are to be connected to J2 and J3 screw terminals in parallel (via respective connectors). For RS-485 interface sensors, polarization resistors are installed. Use of standard interfaces allows one to attach different sensors (not only DO and pH ones) to the measurement module; however, firmware adaptation will be needed due to different register maps of different sensors.

The power system of the module includes a 3.7 V Li-Ion battery, solar cell, and DD05CVSA charger-converter, which allows one to obtain 12 V output and 500 mA peak current. In order to save power, sensors are powered only during measurement on MCU command, and radio module, RS-485 converter, and MCU stay in sleep state between measurement cycles.
